# Stress and Strain Characteristics under the Large Deformation of Surimi Gel during Penetration and Extension Tests Using Digital Image Correlation and the Numerical Simulation Method

**DOI:** 10.3390/gels8110740

**Published:** 2022-11-15

**Authors:** Hwabin Jung, Timilehin Martins Oyinloye, Won Byong Yoon

**Affiliations:** 1 Department of Food Science and Biotechnology, College of Agriculture and Life Sciences, Kangwon National University, 1 Kangwondaehak-gil, Chuncheon 24341, Gangwon, Republic of Korea; 2 Elder-Friendly Research Center, Agriculture and Life Science Research Institute, 1 Kangwondaehak-gil, Chuncheon 24341, Gangwon, Republic of Korea

**Keywords:** surimi, penetration test, ring tensile test, digital image correlation, numerical model, fracture

## Abstract

The stress and strain properties of surimi gels (72.49% moisture content) under large deformation were analyzed during penetration (cylindrical, conical, and spherical puncture) and extension (ring tensile) tests. Mechanical measurements were compared and validated using digital image correlation (DIC) and numerical simulations. The DIC and the finite element method reflected the influence of the probe shape and the surface area in contact with the gel during the measurements. In puncture tests, a larger probe surface increased the strain concentration at the puncture point. In the extension test, the strain distribution was symmetrical. The strain values observed during penetration tests were comparable in both the DIC and numerical simulation. The tensile failure characteristics observed in DIC and numerical simulations are similar to those found in the experiment. The study demonstrated that the extension method with the ring tensile device did not show a stress concentration during the measurement, and DIC and numerical simulation can be effective tools in analyzing the textural properties of surimi gel during the puncture and ring tensile tests.

## 1. Introduction

Surimi is a stabilized fish myofibrillar protein obtained from mechanically deboned fish flesh, which is washed with water and blended with cryoprotectants [1]. It is the main ingredient in surimi seafood, including steamed or fried fish cakes, fish sausage and crab sticks [2]. The most important parameters influencing the texture properties of surimi seafood are the elastic and/or viscoelastic properties of the fish myofibrillar protein gels [2]. Several mechanical tests were used to characterize the texture properties of surimi seafood, such as compression or penetration tests (with cylindrical, spherical, or conical probes) and extension (ring tensile) tests [2,3]. Although the tensile properties of surimi gel are closely related to the sensory result, the penetration test is widely used in the seafood industry because of the simplicity of the measurement process [4].

Generally, the compression or penetration test gives the same information as the tensile test for elastomeric materials [2]. However, since the amount of displacement or strain in a compression or penetration test is limited by the sample height, the test does not fully confirm the force or stress data for large values of displacement or strain, particularly as the strain approaches one (i.e., 1).

In the industry, the mechanical behavior of various materials is evaluated with a tensile test; however, it is difficult to grab food samples for tensile measurement due to stress concentrations and slippage during tensile measurement [5,6]. Park and Yoon [2] developed and successfully measured the tensile properties of fish protein gels using a ring-shaped sample with Laplace’s law. The ring shape of the sample minimizes stress concentration and slippage during tensile measurement so that it can analyze the failure tensile properties of food gels.

Food gel penetration measurements and ring tensile tests may reflect different texture properties due to differences in surface area in contact with the probe and sample during analysis. Thus, a more comprehensive understanding of each texture measurement and its properties will provide a critical interpretation of the texture properties of surimi gel, known as the most important quality attribute of commercial products. It will also describe properties that are similar to the sensory characteristics of the oral cavity. To characterize this, several methods such as digital image correlation (DIC) and numerical simulation have been successfully used along with different texture methods to analyze the textural and viscoelastic properties of foods [2,7,8].

DIC is a deformation measurement method with the advantages of sufficient sensitivity, a simple experimental setup, and a multi-scale view that works by comparing digital images of a component or test item at different strains [2,8]. The method can identify the local strain during the mechanical test, while the mechanical tests only measure the overall strain. Therefore, the DIC technique can provide additional information for comparing mechanical tests, such as penetration and extension (ring tensile) tests. On the other hand, the numerical simulation method offers a powerful design and a nondestructive research tool that can analyze mechanical properties such as food gel stress and strain [1]. The application of numerical simulation in the large deformation properties of a wide range of materials has been discussed extensively in many studies [9,10,11]. In numerical simulation tools, a nonlinear model can simulate and predict the responses of reinforced and pre-stressed material components because each material component exhibits a stress–strain behavior.

Various studies have been carried out to measure the mechanical properties of surimi gel to characterize the influence of different additives on its texture qualities [2,4,12,13]. However, most methods are destructive and limited in the extent of strain. In addition, to understand the effect of different ingredients on the texture of surimi gel, it is necessary first to fully understand how the mechanical properties of the gel change after applying different basic mechanical tests. Therefore, a better understanding of textural methods is needed to select the optimal method for testing the large deformation of surimi gels.

The purpose of this study was to analyze the stress and strain properties of the surimi gel during the penetration (cylindrical, spherical and conical puncture) and extension (ring tensile) tests. The failure point for surimi gel in the extension test was also confirmed by digital image correlation and numerical analysis methods. In addition, the optimal method of measurement between the penetration and extension tests was confirmed.

## 2. Result and Discussion

### 2.1. Mechanical Properties of Surimi Gel

**Figure 1:**
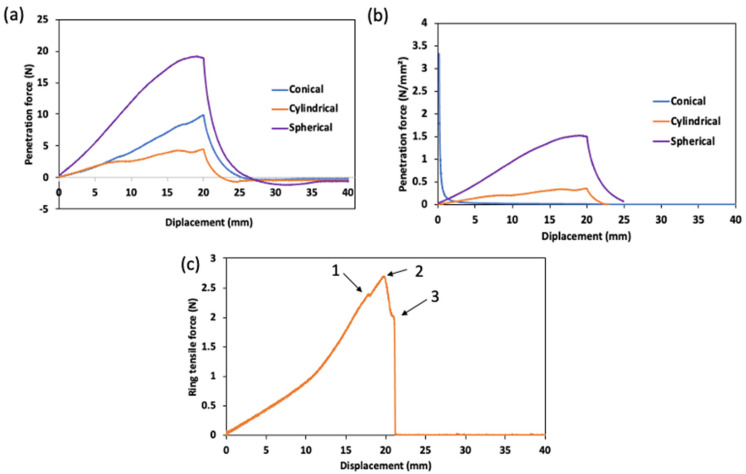
Mechanical properties of surimi gel; (**a**) Force-time curve from the penetration test, (**b**) Normalized penetration force per unit area, and (**c**) Force-time curve from ring tensile until the failure.

The results of the surimi gel’s mechanical properties are presented in Figure 1. During penetration, the probe contacts the sample in both normal and shear directions. Thus, the force detected from the load cell during penetration is a combination of normal and shear force, and the ratio of normal and shear force depends on the geometry (i.e., shape) of the probe and the penetration depth. All force-time curves show breakpoints that reflect the loss of gel structure integrity upon penetration by the probe. More than one breakpoint is observed with the conical probe (Figure 1a). This may be due to the occurrence of many sequential fracture steps. In addition, the force during penetration was directly proportional to the probe surface area (Figure 1a). The measurement with the cylindrical probe showed the lowest penetrating force, which is about three times lower than the value for the conical probe. This may be due to the smaller surface area in contact with the sample compared to other probes. Furthermore, the shape of the penetration probe may also affect the puncture characteristics. In a cylindrical probe, a shear force is generated from the edge and vertical wall of the probe, and thus, the shear force is lower compared to other probes that have an angle of inclination and a significant shear force in the transverse axis.

The influence of the surface area was additionally verified by normalizing the penetration force with the instantaneous surface area of the probe (Figure 1b). Since the normalization of the force per unit area emphasizes the proportion of applied stresses, the differences observed between the probes after normalization should be attributed to the different contributions of shear and normal stresses under or around the probes. The shear contribution is greater at any depth for the conical probes than for the spherical and cylindrical probes. In addition, it increases with the probe depth. Thus, the normalized penetration force decreased sharply with increasing depth for the conical probe, while it increased slightly for the cylindrical and spherical probes. Benedito et al. [14] also reported a strong correlation between puncture strength, probe surface area and probe shape.

The force-deformation curve of the ring tensile test is presented in Figure 1c. As indicated by the shape of the curve, three different stages were identified; (1) is the end of the elastic region defining the yield stress point of the gel, (2) is the end of the strain hardening section which marks the end of plastic behavior, (3) is the failure point during the ring tensile test. The displacement at which the failure occurred in the gel sample was 21.5 ± 1.5 mm. From the mechanics of elastic fracture, the fact that the fracture occurred after the plastic region is interpreted in terms of local stress concentration [15]. The elongation of the gel sample, however, is independent of the exact position from which the deformation occurred but was found to be within 13 ± 4 mm from the bottom pin of the ring tensile probe (result not shown).

### 2.2. Evaluation of Strain and Displacement Properties of Surimi Gel by Digital Image Analysis (DIC)

#### 2.2.1. DIC of a Penetration Test

**Figure 2:**
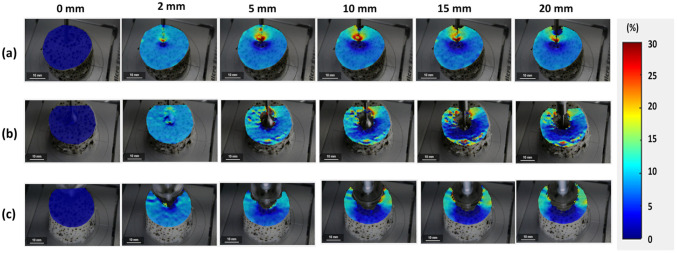
Contour plots of the strain component of surimi gel during a penetration test using (**a**) a cylindrical probe, (**b**) a spherical probe, and (**c**) a conical probe.

Digital image correlation analysis was used to determine the location of the local strain concentration in the surimi gel during the penetration test (Figure 2). In the penetration test, DIC analysis was conducted until the probe reached a depth of 20 mm. Figure 2 shows the increasing strain value with the depth of the probe. It also illustrated a variable strain characteristic that depends on the probe shape. In the cylindrical test (Figure 2a), the strain concentration increased gradually to a depth of 10 mm and then began to decrease. The decrease in the strain concentration was caused by the strain concentration higher in areas closer to the tip of the penetrating probe, and since DIC only analyzes the speckle patterns on the probe surface, it indicates a lower surface strain concentration at a greater probe depth (>15 mm). On the other hand, for the spherical test (Figure 2b), the strain value increased to a depth of 10 mm, after which the concentration became identical to a depth of 20 mm. As the spherical probe mainly compresses the gel due to its spherical shape, it continued to draw the speckle pattern on the gel surface at a constant speed until a depth of 20 mm was reached [14]. In the conical probe penetration test (Figure 2c), the increase in the probe contact area with increasing depth resulted in an increase in the shear force and, thus, a continuous increase in the strain concentration during the penetration test. This result is in line with the penetration force in Section 2.1.

**Figure 3:**
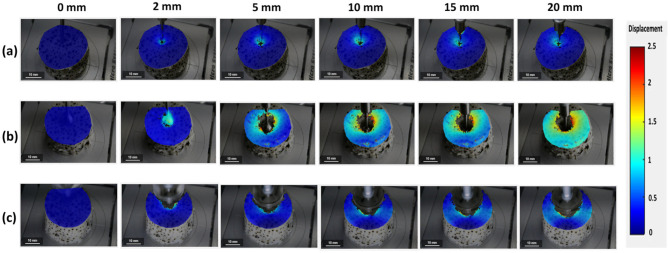
Contour plot of surimi gel displacement during a penetration test using (**a**) a cylindrical probe, (**b**) a spherical probe, and (**c**) a conical probe.

The displacement of surimi gel during the penetration test is presented in Figure 3. As with the strain concentration result, the displacement of the gel sample increased with the probe depth. This is also influenced by the probe’s shape. During penetration tests with a cylindrical probe, the displacement of the gel increased to a depth of 10 mm and then remained constant up to a depth of 20 mm (Figure 3a). The consistent displacement at greater depths (>10 mm) was because of the fact that the deformation in the gel sample was greater in the inner part of the gel than in the surface, which the DIC cannot evaluate. The deformation during the spherical test increased entirely during the analysis (Figure 3b). This is in contrast to the strain concentration, which remained constant between 10- and 20-mm depths. The reason for this observation is that the DIC analyzes material strain based on the location change of the speckle pattern with respect to its reference image; consequently, the strain concentration can remain constant with a short displacement. On the other hand, the displacement is a measure of the movement of the speckle pattern which describes the amount of deformed gel, and since the speckle pattern continued to move due to the increasing shear force during the spherical test, the displacement increased to a depth of 20 mm [2,16]. Furthermore, Figure 3c shows the displacement of the penetration test with a conical probe, which indicates a significantly reduced displacement value compared to the spherical probe. This is related to the narrow cone angle of the conical probe, which allows easy shear penetration. However, after a depth of 10 mm, the transverse axis deformation began to increase. This is related to the shear force exerted by the conical probe’s walls.

#### 2.2.2. DIC Analysis of the Ring Tensile Test

**Figure 4:**
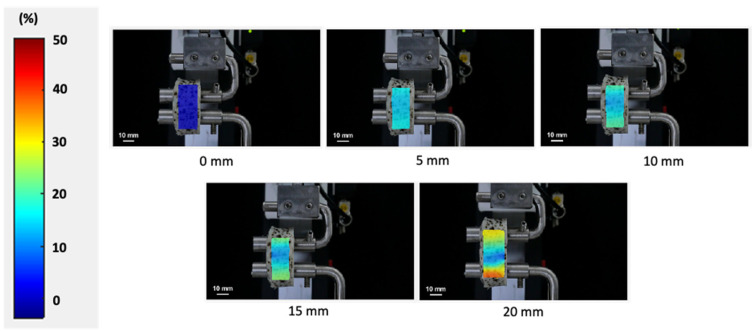
Contour plots of the strain component for surimi gel during the ring tensile test to the failure point.

The local strain of the surimi gel during the ring tensile test analyzed by the DIC method is shown in Figure 4. The ring-shaped gel showed a uniformly increasing strain with raising displacement. For the failure region (area near the bottom pin), no necking phenomenon was observed before the failure. Generally, stress–strain curves for ductile materials such as aluminum and steel were nonlinear with the onset of necking during the ring tensile test, which can be used to estimate the true stress from the neck cross-sectional area [17]. However, for elastic material such as surimi gel, the stress–strain curve is linear without an onset of necking. The uneven local strain around the failure represents the crack propagation before failure. Following this result, it should be emphasized that the surimi gel in this study displayed a prototype tendency towards elasticity rather than ductility. The concentrated local strain at a displacement of 20 mm (near the failure point) caused the gel to break [2]. In addition, the highly concentrated strain at the lower part of the gel may be related to the crack of the surimi gel at this point. As the ring distance increases, the gel material exceeds the linear region, moving through the plastic phase, and thus, the increasing strain around the bottom of the gel may cause the sample to break [17,18,19]. As in our study, Park and Yoon [2] reported that surimi gel without any additives exhibited a similar local strain concentration as the test process approached the failure point. In contrast to the penetration test, which showed a greater strain in the region where the probe penetrates the gel, the ring tensile test showed that local strain increased throughout the area of the sample. Likewise, the maximum local strain in the ring tensile test was higher compared to the penetration test. Therefore, the difference in the relationship between the penetration test (using cylindrical, spherical and conical probes) and the ring tensile test can be explained by the local strain concentration. In addition, the ring tensile test can analyze the stress and strain of the surimi gel up to the fracture point without being affected by the concentration of strain in the sample.

**Figure 5:**
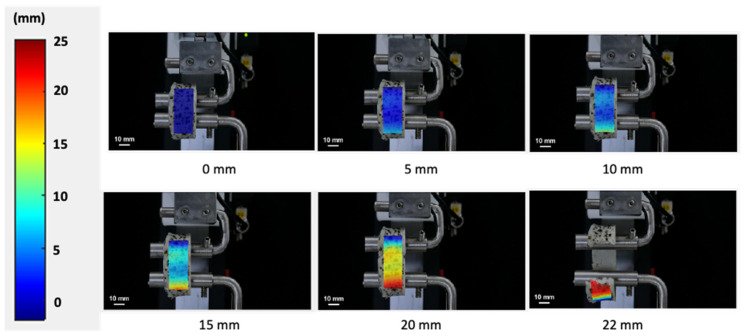
Contour plots of surimi gel displacement during the ring tensile test to the failure point.

DIC displacement analysis was used to further investigate the failure properties of surimi gel during the ring tensile test (Figure 5). Contrary to the strain concentration, which increased steadily from the onset of the tensile test to the fracture in the lower region, the displacement of the gel recorded a lower value until displacement of about 10 mm, after which it rapidly increased, particularly at the fracture zone (near the bottom pin). Generally, the fracture strain is determined by the sample deformation to the point of sudden mechanical yield [20]. In the ring tensile analysis, based on the strain concentration around the fracture position at the failure point, fracture strain was approximately 50%. Compared to the displacement property of the penetration test, the displacement of the ring tensile test increased with increasing textural properties related to surimi gel deformation, while the displacement decreased as penetration distance increased to a depth of about 20 mm, as mentioned in the previous Section 2.2.1 of this study (significantly in a cylindrical probe). It should be noted that our findings suggest that the surimi gel had a high local strain concentration in the penetration test, which could lead to early surimi gel fracture and the instantaneous change could be analyzed until the probe reaches a particular depth. In the studies previously reported for the texture of meat and meat analogues, the puncture measurement method may not be reliable due to the narrow penetration part, which may not be able to represent the whole product, especially when the product has a heterogeneous nature [21]. However, in the ring tensile test, the strain could be analyzed up to the end of the test (i.e., the failure point). In addition, the strain is applied to the whole specimen unlike puncture test. Tensile tests have also been used in several food products such as sausages and meat, etc., for which authors reported that the tensile test was an effective measurement method to analyze the resistance of the product to tearing or fracturing [22,23]. Thus, utilizing the DIC, non-contact approach, it would be feasible to quantify and compare the local strain concentration from the image data, provided the design of the probe or test generates significant stresses on the gel sample surface, and in this study, tensile test was proven to be a better measurement method for surimi gel than the penetration test. DIC has recently been used in a variety of industries to quantify strain quality [24,25,26].

### 2.3. Evaluation of Stress and Strain Properties of Surimi Gel by Numerical Simulation

**Figure 6:**
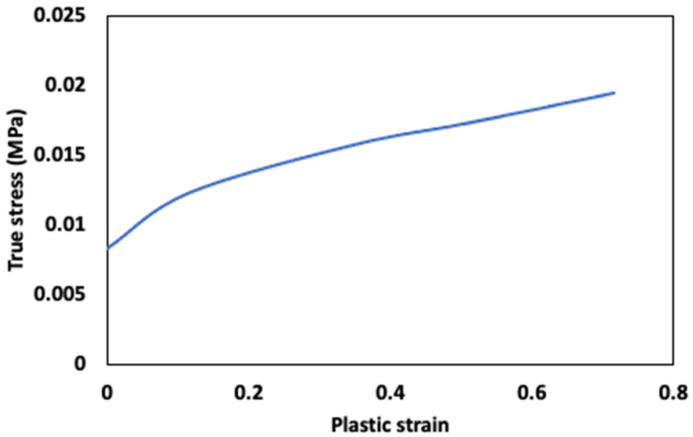
Curve of surimi gel true stress vs. plastic strain.

The material properties and Johnson–Cook parameters described in Equation (8) used to simulate the penetration and ring tensile are presented in Table 1 and Figure 6. The fit of the data was obtained in the ANSYS software.

#### 2.3.1. Numerical Analysis by a Penetration Test

**Figure 7:**
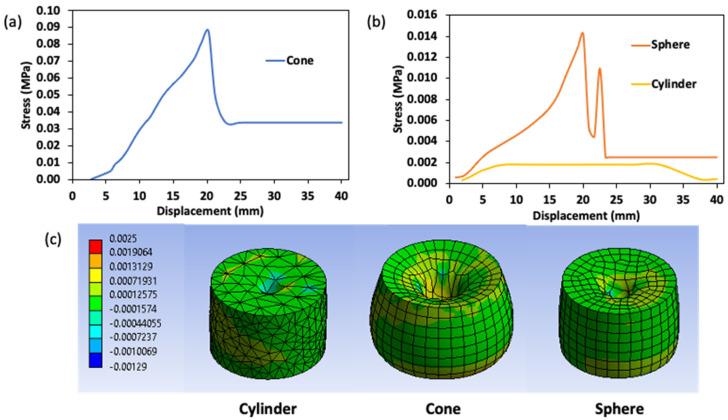
Stress curve of various penetration probes during puncture analysis for surimi gel; (**a**,**b**) quantitative stress value and (**c**) contour description of the stress distribution in a puncture gel at a depth of 20 mm.

The stress curve of various penetration probes during the surimi gel puncture analysis is presented in Figure 7. The von Mises criterion was applied to estimate the total contact stress, which was defined as the sum of the shear and normal stress components in the gel sample [27]. Overall, the simulation model was able to distinguish changes in the true failure stress experienced by the gel samples at various failure distances (i.e., probe displacement). It was also possible to unequivocally characterize the impact of the penetration probe shape. As shown in Figure 7a,b, the stress was dispersed from the moment the probe tip touched the sample and generated a zone of increasing stress as the probe penetrated the gel’s surface. The impact of the probe penetrating the gel created a sequence of stress curves during puncture. With the exception of the cylindrical test, which showed an increase in stress value to about 8 mm (0.02 MPa) and then kept constant; these stress curves increased sharply to a depth of 20 mm and were highest in the conical probe (0.09 MPa). This may be due to the lower shear force on the walls and edges of the cylindrical probe compared to the walls of other penetration probes with an angle of inclination that increases the shear stress in the transverse axis. The reduction in stress value from a depth of 20 mm in the conical and spherical probes shows the return of the penetration probe to its starting position after completing a 20 mm puncture. At this point, the influence of the probe shape became apparent (Figure 7a). The return stage of the spherical probe created a second peak stress (0.011 MPa) due to the adhesion effect caused by the upper parts of the spherical probe that drags along the gel sample.

**Figure 8:**
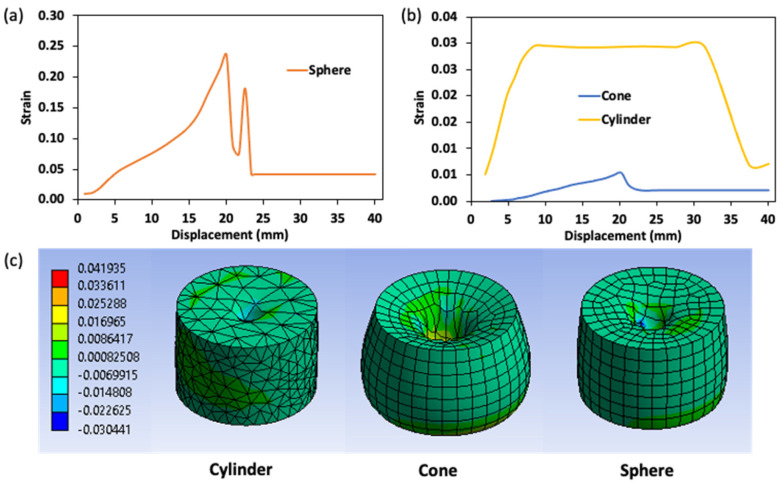
Strain curve of various penetration probes during puncture analysis for surimi gel; (**a**,**b**) quantitative strain value and (**c**) contour description of the strain distribution in a puncture gel at a depth of 20 mm.

The true strain–displacement curve is shown in Figure 8. The strain curve has a similar shape to the stress curve. However, the strain value is greatest when measured with a spherical probe (0.245) (Figure 8a). This result is identical to the value observed during the DIC study and is related to the spherical probe design which lowered the penetration ability, in particular, compressing the gel sample more than the other probes, therefore significantly increasing the strain properties of the gel. In this regard, the conical probe demonstrated the lowest peak strain (0.01) due to its narrow angle, which facilitates gel penetration (Figure 8b).

#### 2.3.2. Numerical Analysis of the Ring Tensile Test

**Figure 9:**
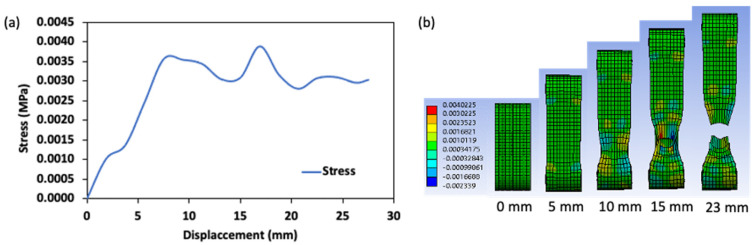
Stress distribution in surimi gel during the ring tensile test; (**a**) quantitative stress value and (**b**) contour description of the stress concentration region.

**Figure 10:**
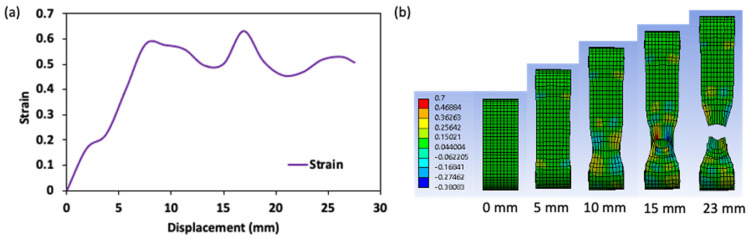
Strain distribution in surimi gel during the ring tensile test; (**a**) quantitative strain value and (**b**) contour description of the strain concentration region.

Figure 9 and Figure 10, illustrate the stress and strain characteristics of a simulated ring tensile test. The ring tensile stress increased uniformly with the gel elongation to about 8 mm displacement (i.e., top pin displacement), and then shifted in a sinusoidal form with a stress value ranging from 0.003 to 0.0043 MPa until failure occurred at a displacement of 20.5 mm (time = 41 s). It is related to plastic characteristics. The onset of plastic deformation does not reflect the complete failure of the gel but rather the onset of the strain-softening stage, as indicated by the unstable stress distribution [28]. The maximum stress was detected around the bottom pin of the ring tensile probe, as shown in Figure 9b. The area of the maximum local stress concentration at the failure point resulted from the crack of the gel sample caused by the stress concentration. This reduces the gel resistance to plastic deformation [29,30]. In addition, the elastic properties of the gel decreased, and the local stress became high, showing a weaker ability to release concentrated stress from the region near the bottom of the ring to the area in the top position. The gel strain characteristics during the ring tensile test also showed a similar flow curve as the stress curve (Figure 10a). Here, a uniformly distributed strain concentration was recorded as the elongation process approached the failure point when the strain increased significantly in the region near the bottom pin. The high concentration increased from approximately 10 mm of displacement. The increase in strain during the plastic phase led to the crack in the transverse width of the ring tensile sample [18,19]. In addition, the high-strain region found by the simulation result agrees with the strain result in the DIC analysis for this study. Thus, both DIC and numerical methods can be effective tools in analyzing the stress and strain properties of surimi gel by ring tensile analysis.

## 3. Conclusions

In this study, the stress and strain distribution was assessed during puncture tests (using cylindrical, spherical and conical probes) and the surimi gel ring tensile tests using DIC and numerical simulation (i.e., the Johnson–Cook model). The normalized penetration force of the surimi gel revealed the influence of the probe’s surface area in contact with the sample, as well as the effect of the probe shape. The conical probes with increasing surface area during puncture had a maximum penetration force that decreased with increasing puncture depth due to an increase in shear force. The high shear force is because of the shape and inclination angle of the probe. The DIC successfully analyzed the strain characteristics from the images obtained during the puncture and the ring tensile tests, revealing the influence of the probe surface area and shape. The strain characteristics observed during the penetration and ring tensile tests were comparable to those obtained during the DIC analysis. During DIC and numerical simulation, the ring tensile test revealed a failure point with a displacement of 22 mm and 20 mm, respectively. This range is close to the experimental result (21.5 ± 1.5 mm). Both the DIC and the numerical simulation revealed the limitations in analyzing the stress and strain characteristics of the surimi gel with the penetration test due to a significant increase in the strain and stress concentration in the probe contact region. These limitations do not exist for the ring tensile test, which is able to uniformly distribute stress and strain in the sample, allowing the properties of the gel sample to be analyzed up to the failure point. Based on this study, the ring tensile test is the most appropriate method to analyze the texture characteristics of surimi gel and can be confirmed by both the DIC method and the numerical method.

## 4. Materials and Methods

### 4.1. Surimi gel Preparation

High quality (FA grade) Alaska pollock (Trident Seafood, Seattle, WA, USA) with a moisture content of 75.34% and a pH of 6.8 was provided by Pulmuone Co., Ltd. (Gwangpyeong-ro, Seoul, Republic of Korea). Frozen surimi was thawed at room temperature (25 ± 2 °C) for 4 h before cutting into cubes (3 cm^3^). Following the description of Park and Yoon [2], sodium chloride 2 g/100 g surimi paste was added prior to the chopping process. The surimi cubes were chopped with a universal food processor (Model UMC5, Stephan Machinery Corp., Hameln, Germany) at low speed for 2 min. By continuously circulating the ice water (0 °C) around the universal pan of the food processor, the sample temperature was kept <5 °C. The paste was stuffed into stainless steel cylinders (diameter = 50 mm; length = 30 mm). The cylinders were heated in a water bath at 90 °C for 30 min. Boiled gels with a moisture content of 72.49% were quickly chilled in ice water (0 °C). The gels were refrigerated at 5 °C overnight prior to analysis.

### 4.2. Mechanical Analysis of Surimi Gel

#### 4.2.1. Penetration Test

The penetration test was performed using a TA-XT texture analyzer (Stable Micro Systems, Surrey, UK) equipped with a spherical (Taylor) plunger (Model: TA-18 with ball diameter 4 mm), a cylindrical probe (Model: TA-54 with diameter 4 mm and height 35 mm), and a conical probe (Model: TA-2 with a base diameter of 12.66 mm and a cone angle of 60°). Cold surimi gels (5 °C) were placed at room temperature for 2 h before testing. The penetration analysis conditions were set as a test speed of 1 mm/s and a travel distance of 20 mm down into the gel such that approximately 67% of the total gel height is punctured by the large deformation. The instantaneous penetration force (N) as a function of displacement was recorded. All measurements were conducted 10 times.

#### 4.2.2. Ring Tensile Test

**Figure 11:**
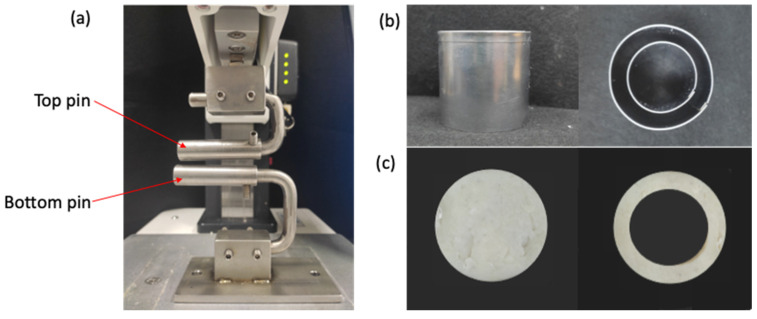
Schematic representation of the ring tensile test system (**a**), stainless steel stamp for the preparation of ring-shaped surimi gel (**b**), and the cylindrical and ring sample for the penetration and ring tensile test (**c**).

According to Park and Yoon [2], the failure ring tensile test was performed with a TA-XT texture analyzer equipped with two pins (diameter = 10 mm) (Figure 11a). In this test, the cylindrical gels were cut to a disk shape (Outer diameter = 40 mm, inner diameter = 35 mm, ring width = 5 mm and height = 20 mm) (Figure 11b,c). In a tensile test, samples are usually deformed uniaxially along the long axis of the sample until failure occurs. During the tensile analysis, the top pin of the device was set to move up (displacement = 50 mm) to break the ring-shaped gel under a tensile force at a constant speed of 0.5 mm/s. The tensile test result is recorded as load or force versus elongation. The load and elongation are normalized to the appropriate engineering stress and strain parameters. All experiments were conducted 10 times.

### 4.3. Mechanical Properties of Surimi Gel

The mechanical properties of surimi gel were determined using the method described by Park and Yoon [2]. The ring tensile analysis was conducted as presented in Section 4.2.2. The tensile force was applied to the surimi gel until the failure of the sample. The force-displacement data from the ring tensile test was used to calculate the engineering stresses and strains using Equations (1) and (2),

(1) $$\sigma_{eng}=\frac{F}{wH}$$

(2) $$\varepsilon_{eng}=\frac{\Delta L}{L_{0}}$$

where *σ_eng_* (MPa) and *ɛ_eng_* are, respectively, the engineering failure ring tensile stress and strain; *F* is the load (N) measured during the ring tensile testing; *w* is the width of the ring specimen (m); *H* is the height of the gel (m), *L* is the instantaneous length extension (m), and *L*_0_ (m) is the initial ring length. In engineering practice, the mechanical properties of materials are usually described by engineering stress–strain curves. However, in numerical simulation, material properties are commonly described with true stress–strain curves [31]. In the uniaxial tension test, the true stress (*σ_true_*) and true strain (*ɛ_true_*) are calculated from Equations (3) and (4), respectively.

(3) $$\sigma_{true}=\sigma_{eng}\left(1+\varepsilon_{eng}\right)$$

(4) $$\varepsilon_{true}=\ln(1+\varepsilon_{eng})$$

Information from true stress (*σ_true_*) vs. strain (*ɛ_true_*) was used to determine Young’s modulus (*E*), which is represented as the slope of the linear region before the yield stress point. For the numerical analysis, the region of plastic deformation in the stress–strain curve was used to analyze the elastic (*ɛ_elastic_*) and plastic (*ɛ_plastic_*) strain using Equations (5) and (6), respectively,

(5) $$\varepsilon_{elastic}=\frac{\sigma_{true}}{E}$$

(6) $$\varepsilon_{plastic}=\varepsilon_{Total}-\varepsilon_{elastic}$$

where *ɛ_Total_* is the total strain.

### 4.4. Image Analysis

#### 4.4.1. Digital Image Correlation (DIC)

DIC is used as a reliable tool in experimental mechanics to obtain displacement and strain of the entire field [2]. It is based on the image comparisons of samples covered with randomly sprayed black dot speckle patterns. The speckle pattern of the undeformed sample (reference image) is compared with the images of the deformed sample. The displacement is then calculated using the pattern-matching principle. The speckle patterns in this study were created manually by spraying black ink (Pelikan 4001, Pelikan Inc., Schindellegi, Switzerland) onto a gel sample. As it was impractical to compare each pixel in the picture, a small region comprising numerous pixels (subsets) and the region in contact with the test probe were approximated. Pattern matching was based on obtaining the highest correlation between image subsets in the undeformed and deformed states. Sequential frames captured at different time intervals were used for two-dimensional DIC analysis in an open-source program (Ncorr v1.2) with MATLAB (Mathworks^®^ Inc., Natick, MA, USA). A detailed description of the methods applied to assess the local strain can be found in Blaber et al. [16].

#### 4.4.2. Image Acquisition

**Figure 12:**
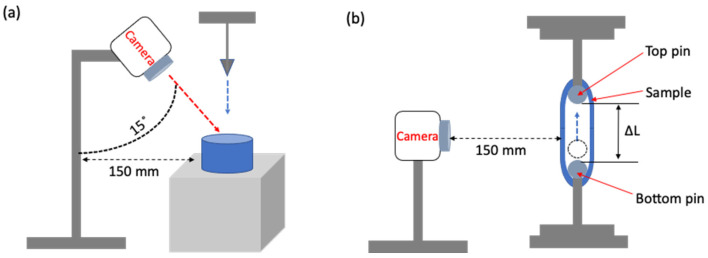
Schematic diagram of an image acquisition system for (**a**) penetration test, and (**b**) ring tensile test.

To analyze the instantaneous changes in the speckle pattern during the penetration and ring tensile tests, an image processing technique was used, which includes (1) video recording, (2) image frame acquisition, and (3) image frame analysis based on the video time stamp. A digital camera (DSLR-500D, Canon Inc., Tokyo, Japan) was used in each test to record video at frame 30 fps and a resolution of 2.07 million pixels. For the penetration test, the camera was positioned to record the penetration point of the surimi gel at an angle of 15° and a distance of 150 mm from the gel (Figure 12a). In the failure ring tensile test, the camera was placed horizontally 150 mm to record the front view of the gel sample (Figure 12b). In order to analyze several frames from the video, the following conditions were set; (1) the first frame (i.e., reference image) is the frame at the point when the probe contacted the sample, (2) the last frame (i.e., deformed image) is the frame at the point when puncture was completed in the penetration test, and the fracture occurred in the ring tensile test, and (3) the frame of instantaneous deformation between the first and last frames was collected every 30 frame intervals. The analysis was conducted on at least five different videos recorded for each test.

### 4.5. Numerical Analysis

Ansys 2020 R2 with Explicit dynamic module (Ansys, Inc., Canonsburg, PA, USA) was used to characterize the penetration effect and the failure point for the surimi gel in a penetration test (i.e., using a cylindrical, spherical and conical puncture) and the ring tensile test, respectively.

#### 4.5.1. Geometry Description

**Figure 13:**
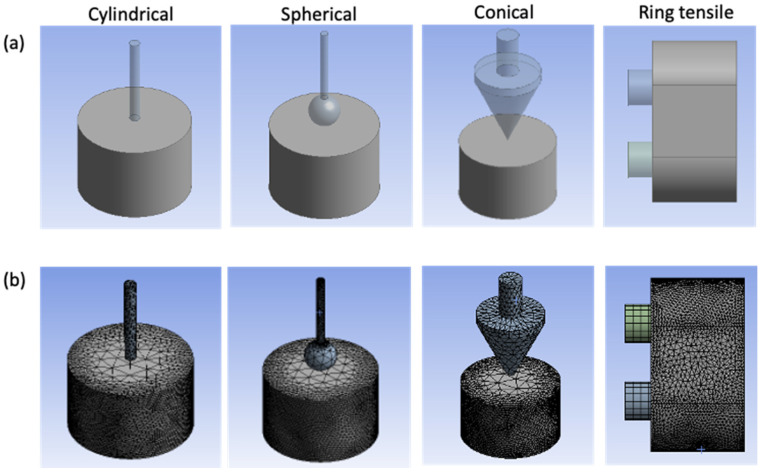
Geometry description of surimi gel during numerical analysis (**a**), and mesh model (**b**).

The sample geometry design was completed in the ANSYS workbench 2020 R2. The dimensions of the surimi gel and probes are the same as described in Section 4.2.1 and Section 4.2.2 for mechanical analysis (Figure 13a). A triangular/tetrahedral mesh was used to discretize the fracture domain in geometry. On the curved faces, for both the cylindrical and ring tensile model, a level 3 mesh refinement was applied to accurately capture the stretch and fracture properties (Figure 13b). A total of 16,191 to 18,031 and 17,138 elements were generated for the penetration and ring tensile analysis, respectively.

#### 4.5.2. Finite Element Method (FEM) Simulation

The Johnson–Cook model dependent on temperature and strain rate was used for the FEM simulation [32,33]. In this case, the true stress is described by Equation (7):

(7) $$\sigma_{true}=\left(A+B{\left(\varepsilon_{plastic}\right)}^{n}\right)\left[1+C\;ln\frac{{\dot{\varepsilon }}_{plastic}}{{\dot{\varepsilon }}_{0}}\right]\left(1-{\left(\frac{T-T_{material}}{T_{melt}-T_{material}}\right)}^{m}\right)$$

where *σ_true_* is the true stress, *A* is the initial yield strength of the surimi gel at room temperature (MPa), *B* is the hardening modulus (MPa), *ɛ_plastic_* is the true plastic strain, *n* is the strain hardening exponent, *C* is the parameter representing the strain rate sensitivity, ${\dot{\varepsilon }}_{plastic}$ is the true plastic strain rate (s^−1^), ${\dot{\varepsilon }}_{0}$ is the reference strain rate (s^−1^), *m* is the parameter evaluating the thermal softening effect, *T* is the temperature (°C), *T_melt_* is the melting point temperature (°C), and *T_material_* is the material transition temperature (°C). Since the influence of the temperature change due to the friction of the probe against the gel sample was not considered, the general form of the model in Equation (7) becomes Equation (8).

(8) $$\sigma =\left(A+B{\left(\varepsilon_{plastic}\right)}^{n}\right)\left[1+C\;ln\frac{{\dot{\varepsilon }}_{plastic}}{\varepsilon_{0}}\right]$$

A failure criterion is required to characterize the gel material properties degradation due to the probe penetration or the tension from the ring tensile probe. The Johnson–Cook failure model based on plastic strain was used. In this model, the failure occurs when parameter *D* becomes 1:

(9) $$D=\int \frac{1}{\varepsilon_{f}}d\varepsilon_{plastic}$$

where *ɛ_f_* is the true fracture strain. The true fracture strain is given by Equation (10)

(10) $$\varepsilon_{f}=\left(d_{1}+d_{2}exp^{-d_{3}\frac{\sigma_{m}}{\sigma }}\right)\left[1+d_{4}ln\frac{{\dot{\varepsilon }}_{plastic}}{\varepsilon_{0}}\right]\left(1+d_{5}\right)$$

where *d*_1_ to *d*_5_ are the material damage model constants obtained by the MATLAB curve fitting tool as *d*_1_ = 0.35, *d*_2_ = *d*_3_ = *d*_5_ = 0.001, *d*_4_ = 0.021, and *σ_m_* is the mean stress (MPa).

The boundary conditions were set as follows: in the penetration analysis, the bottom of the cylindrical gel was fixed, and the top of the puncture probe was assigned a total displacement of 20 mm, which translated along the negative (–) *z*-axis at a displacement rate of 1 mm/s. In the ring tensile analysis, the bottom pin was fixed (stationary), and the top pin was assigned a 50 mm displacement along the *z*-axis at a displacement rate of 0.5 mm/s.

One of the main challenges encountered during the simulation was excessive mesh distortion and failure in the convergence criteria. Therefore, an explicit finite element code based on the adaptive mesh technique was used, which allows the mesh to automatically regenerate when elements are distorted due to large deformations. In an explicit model, an adaptive mesh generates a new mesh and remaps the solution parameters from the old mesh to the newly generated one. In this study, adaptive meshing was performed for every three probe increments, with five mesh sweeps per adaptive mesh increment. Additionally, in order to shorten the computational time, the mass scaling technique was used, which modifies the densities of materials in the model and improves the computational efficiency [30]. The mass scaling used in this study was performed every 10 increments to obtain a stable time gain of at least 0.0001 s step time.

### 4.6. Statistical Analysis

We characterized at least five freshly prepared gel samples for each analysis. Mean values and standard deviations were calculated for each measurement. Data were analyzed using the statistical analysis software SPSS 19.0 and results were reported as mean values ± standard deviations.

## Figures and Tables

**Table 1:** Surimi gel properties and Johnson–Cook parameters.

Parameter	Value
Young modulus, *E* (MPa)	0.01719
Poisson’s ratio	0.5
Density, *ρ* (g/cm^−3^)	0.00097
Initial yield strength, *A* (MPa)	0.00833
Hardening modulus, *B* (MPa)	0.01212
Strain hardening exponent, *n*	0.6752
Strain rate constant, *C*	0.07
Reference strain rate, *ɛ*_0_ (1/s)	1

## Data Availability

The data presented in this study are available on request from the corresponding author.
